# Production of Low‐fat mayonnaise without preservatives: Using the ultrasonic process and investigating of microbial and physicochemical properties of the resultant product

**DOI:** 10.1002/fsn3.2227

**Published:** 2021-03-08

**Authors:** Rojin Tavakoli, Mostafa Karami, Samira Bahramian, Ario Emamifar

**Affiliations:** ^1^ Department of Food Science and Technology Sanandaj Branch Islamic Azad University Sanandaj Iran; ^2^ Department of Food Science and Technology Bu‐Ali Sina University Hamedan Iran

**Keywords:** mayonnaise, preservatives, scanning electron microscopy, shelf life, ultrasonication

## Abstract

In this study, ultrasonication was used at 20 kHz, 750 W for 5 min, as a nonthermal alternative to pasteurization and as a substitute for benzoate‐sorbate preservatives. Also, its efficiency on microbial and physicochemical properties of low‐fat mayonnaise stored at 4°C was investigated. The results showed the reduction of total counts of micro‐organisms, acid‐tolerant bacteria, molds, and yeasts during six months shelf life compared with the control samples. Sonicated mayonnaise samples had lower pH values and higher acidity in comparison with control samples during the storage. The speculation was verified through the microstructure of mayonnaise samples during storage time observed by *SEM* micrographs. The overall results indicated that it was possible to produce sodium benzoate and potassium sorbate‐free mayonnaise using the ultrasonic nonthermal method.

## Graphical abstract

- In this study, Ultrasonication was replaced with traditional preservatives of low‐fat mayonnaise.
- Shelf life of low‐fat mayonnaise increase with its ultrasonication.
- Molds, yeasts, and other spoiling micro‐organisms can be controlled with ultrasonication
- Micrographs of *SEM* can be successfully used for texture changes of sonicated low‐fat mayonnaise

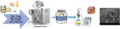

## 1 INTRODUCTION

Recently, food manufacturers trying to use the labels on their products to claim that they can use natural ingredients with no chemical additives in the formulation of their products, so that they can gain customer trust (Aganovic et al., 2018). Benzoic acid (E210), sorbic acid (E200), and their salts are used as preservatives in the formulation of many foods and drinks (Mischek & Krapfenbauer‐Cermak, 2012; Piper & Piper, 2017). Semisolid mayonnaise (oil in water emulsions) is one of the food products which has a notable demand in the market (Alvarez‐Sabatel et al., 2018; Chivero et al., 2016). The market opportunities for this segment are on the rise and expected to grow annually by 2.6% from 2018 to 2021 (Sauces & Condiments, 2018). The average content of sodium benzoate and potassium sorbate is below 750 mg/kg established by the Iranian National Standard Organization (ISIRI), for mayonnaise (ISIRI, 2008). Previous studies demonstrated that the benzoic and sorbic acid have the risk to undergo a transform to potential mutagens. Chromosome disorders, DNA damages, and pseudo‐allergy in sensitive patients and hyperactivity in children have been reported due to the consumption of these compounds (Mamur et al., 2012; Piper & Piper, 2017; Pongsavee, 2015). Pregnant women should avoid consuming the foodstuff containing sodium benzoate as an additive because their Lead content showed to cause genomic damages in the liver cell samples of pregnant rats and their fetuses (Saatci et al., 2016). Sodium sorbate could form genotoxic and cell‐transforming agents, such as 1,4‐dinitro‐2‐methyl pyrrole and ethyl nitrolic acid under some conditions such as low pH, heating, and storage. The Joint FAO/WHO Expert Committee on Food Additives (JECFA) has investigated the safety of these compounds (Mischek & Krapfenbauer‐Cermak, 2012; WHO, 2016). Therefore, the Acceptable Daily Intakes (ADIs) have reported: 0–5 mg/kg body weight/day for benzoic acid (and benzoate salts) and 0–25 mg/kg body weight/day for sorbic acid (and sorbate salts) (Chaleshtori et al., 2018; Mischek & Krapfenbauer‐Cermak, 2012). Sodium benzoate and potassium sorbate content of 103 food and drink samples in Kashan (Iran) with an estimation of human health risk for the Iranian population was analyzed (Chaleshtori et al., 2018). They reported chronic daily intake (CDI) of sodium benzoate per day for mayonnaise sauce and carbonated soft drink products were much higher than ADI (5 mg/kg BW/day). Their findings indicated that the target hazard quotient (THQ) and hazard index (HI) values of sodium benzoate for mayonnaise sauce and carbonated soft drink products were more than 1 mg/kg BW/day through consumption of these products which are at a harmful level. According to the estimated HI values and the potential health risk hazard to exposed population, the possibility of replacing sodium benzoate and potassium sorbate with plant essential oils or nonthermal pasteurization methods such as pulsed electric field, high‐pressure homogenization, high hydrostatic pressure, and ultrasound can be considered as a viable alternative (Mischek & Krapfenbauer‐Cermak, 2012; de Souza et al., 2016). Production of a healthy mayonnaise is a concern in the food science and technology, so that some researchers proposed new functional mayonnaise with addition of probiotics, antioxidants, and prebiotics (Mirzanajafi‐Zanjani et al., 2019). Some other researchers used natural preservatives to produce sorbate‐benzoate free mayonnaise with addition of mustard paste, annatto, and nanoliposomes (NLs) containing the phenolic compounds of pistachio green hull (Rafiee et al., 2018).

Ultrasound is a green technology and one of the new preservation techniques. It can eliminate microbial activity, rupture cells, and denature enzymes, while keeping nutritional value and organoleptic characteristics (texture, color, and taste). The process has some benefits such as accelerating the rate of food sterilization, significant energy savings, a competitive cost, environmental friendliness, a high degree of safety, and greater homogeneity. The mechanism of the antimicrobial function is thinning the cell membranes and disrupting cell walls of microbes, localized heating, and production of free radicals (Wang et al., 2020). The inactivation effect of ultrasound is related to the generation of intracellular cavitation and these mechanical shocks can disrupt cellular structural and functional components up to the point of cell lysis (Chemat et al., 2017). The reaction of different micro‐organisms to ultrasound treatment is different. The factors that affect the effectiveness of microbial inactivation are the amplitude of ultrasound waves, exposure or contact time, the volume of processed food, the composition of food, and treatment temperature. The performance of the above‐mentioned factors is also affected by the type, shape, or diameter of the micro‐organisms. Bigger cells are more sensitive than small ones. This susceptibility is probably due to their larger surface area. Gram‐positive bacteria are known to be more resistant than gram‐negative ones, possibly because of their thicker cell wall, which provides better protection against ultrasound effects (Drakopoulou et al., 2009). Ultrasonication has many beneficial effects on food processing, so that recently some researches have been studied about the application of ultrasonication, solely or in combination with other methods, on baked products (Esmaeilzadeh‐Kenrari & Nemati, 2020), fruit juices (Belgheisi & Esmaeilzadeh‐Kenrari, 2019; Yu et al., 2020), reduced‐sodium bacon (Zhou et al., 2020), optimization of ultrasound‐assisted enzymatic extraction (Li et al., 2020) and bioactive compounds (Fernandez‐Barbero et al., 2019), stabilization of raw milk (Scudino et al., 2020), and whey‐beverage (Guimaras et al., 2019).

One of applications of ultrasonication is acoustic emulsification that offers the following improvements over conventional methods. The produced emulsion has particles in the sub‐micron range with an extremely narrow particle size distribution, and the resultant emulsions are more stable. Aganovic et al. (2018) described a new technique for the preparation of mayonnaise by ultra‐high‐pressure homogenization and stabilization of emulsions by reducing the size of oil droplets and improving interactions between emulsifier and fat phase. The ultrasound field produces propagating waves on the oil–water interface that subsequently become unstable. These waves result in the eruption of the oil phase into the continuous aqueous phase (in a typical oil‐in‐water emulsification system), leading to the formation of medium to large droplets. Also, ultrasonication produces a series of instant compression and expansion waves (Zhang, Liao, et al., 2020). This mechanism continues to reduce the droplet size once a coarse emulsion is formed (Leong et al., 2018). In situ *SEM* observation and analysis are a beneficial strategy to display the mechanism of material failure (Leong et al., 2018; Ma et al., 2019; Zhang, Chen, et al., 2020). Also, the chemical distribution revealed by confocal laser scanning microscopy and (cryo‐*SEM*) images can be a new approach to indicate mayonnaise microstructure (Bi et al., 2019). The dispersive nanoparticles from ultrasonic‐treated casein protein evidently were absorbed on the interface of Pickering high internal phase emulsions (HIPEs). These findings prove that ultrasound is a useful tool to loosen casein flocs to induce the in situ formation of stabilized Pickering HIPEs (Zhou et al., 2018). Also, Tavakoli et al. (2021) indicated that about consumer acceptance, ultrasonicated preservative‐free mayonnaise had acceptable sensorial attributes such as texture, odor, and taste, and the ultrasonication had no adverse effect on sensorial properties of mayonnaise.

Therefore, a better understanding of physicochemical and microbial changes of mayonnaise during ultrasonic processing and shelf life would be performed. Also, the effects of ultrasonic on the emulsifying properties of mayonnaise would provide valuable information for food manufacturers to broaden the utilization of this technique to made emulsions. To our knowledge, such information is lacking in scientific literature and warrants further evaluations. Thus, this study was performed in a low‐fat mayonnaise to determine the effect of ultrasound processing compared with conventional preservatives on the survival of micro‐organisms during the storage time at 4°C. The mechanism of the cavitation erosion behavior of the mayonnaise was investigated using in situ *SEM* observation after different periods.

## 2 MATERIALS AND METHODS

### 2.1 Laboratory preparation of mayonnaise

All of the preparation procedures for low‐fat mayonnaise were carried out in R&D section of Sahar® Food processing company. Mayonnaise samples were prepared in a laboratory‐scale vessel homogenizer (Pilot vacuum homogenizer, Parsian Tarh Sina Co., Iran). The following ingredients (Table 1) were used to make mayonnaise throughout the study and based on the usual industrial procedure early described by Tavakkoli et al. (2021):

**TABLE 1:** Percentage recipes of preservative‐free mayonnaise (wt., %)

Ingredients	Weight (%)
Tap water	59.76
Soy oil	21
Pasteurized whole egg	1.47
Sugar	4.65
Salt	1.9
Mustard	0.5
Vinegar	5
Citric acid powder	0.13
Guar	0.5
Carboxymethylcellulose	0.5
Xanthan	0.2
Sorbate potassium	0
Starch	3.91
Sodium benzoate	0
Lemon juice	0.48

Tap water (59.76%), soy oil (21%) (Naz‐Gol edible oil, Iran), pasteurized whole egg (Narin, Hamedan, Iran) (1.47%), sugar (4.65%) (Hegmataneh Sugar refinery Co., Hamedan, Iran), salt (1.9%) (Ultra‐pure, Pegah Malayer, Iran), mustard (0.5%) (Niusheh, Hamedan, Iran), vinegar (5%) (Takestan, Iran), dry citric acid powder (0.13%) (Magnolia, Iran), guar (0.5%) (AbdolGhader, India), carboxymethylcellulose (0.5%) (Glucosan, Ghazvin, Iran), xanthan (0.2%) (Fufeng, China), potassium sorbate (0%) (Jahan‐Shimi, Iran), starch (3.91%) (Glucosan, Ghazvin, Iran), sodium benzoate (0%) (Merck, Darmstadt, Germany), and lemon juice (0.48%) (Urum‐Ada, Urmia, Iran). In the control samples, 0.37% potassium sorbate and 0.37% sodium benzoate were added. First, all of powdered ingredients mixed, then vinegar and half of the oil were added and during 13‐min mixing, the second half of the oil was added. The above‐mentioned procedure was real formulation and production instructions for low‐fat mayonnaise in Sahar® food processing company (Hamedan, Iran).

Concentrated mayonnaise‐like systems were made in 5 kg batches for each treatment. At least three replicates of each treatment were prepared. The whole production process was under full hygienic conditions, that is, cleaning and disinfecting of the equipment.

### 2.2 Ultrasound treatment

Mayonnaise samples (250 ml) sonicated using an ultrasonic homogenizer (JY92‐IIDN, Co. Ltd., Ningbo, China) fitted with an autoclavable 13.5 mm diameter titanium probe which was dipped into mayonnaise with a depth of 50 mm, (20 kHz, 750 W, and 0.3 W/cm^3^) for 5 min (pulse duration of on‐time 2s and off‐time 2s). Ice was added into the ice‐water bath to maintain the temperature of the mayonnaise below 20°C, which actually was maintained at 14–18°C during the process. Before and after each experiment, the ultrasound probe was sterilized by rinsing with ethanol 70%. The last part of mayonnaise, without ultrasonic treatment, was control sample. The control groups were consisted of the samples with or without added preservative. The produced mayonnaise samples were stored at 4°C until further analysis.

### 2.3 Micro‐organisms determination

The total number of colonies (total micro‐organisms count), mold, yeast, acid‐tolerant micro‐organisms, *Escherichia coli*, and heterofermentative lactic acid bacteria of samples were enumerated and reported according to the National Standard of Iran (ISIRI, 2017). According to ISIRI (2017), the total micro‐organisms count, molds, yeasts, and acid‐tolerant micro‐organisms must be less than 10^4^, 10^2^, 10^2^, and 10^3^ CFU/g, respectively, with deficiency of *E scherichia coli* and heterofermentative lactic acid bacteria (lack). At first, for serial dilution, suspensions were prepared by the Ringer solution. Samples (5g) were added into (45cc) sterile Ringer solution, in a 400‐ml stomacher. The bags were homogenized for 2 min at 8,000 rpm (SH‐IIM, Anke Biotechnology Co., Shanghai, China). A series of 10 times dilutions in Ringer solution was made and cultured using pure plate method with Plate Count Agar medium (Merck, Darmstadt, Germany), then incubated at 37°C for 72 hr for total number of colonies count (ISO4833‐1, 2013), incubated at 25°C for 120 hr in dicholoran (18%)‐glycerol (DG18) culture medium (Merck, Darmstadt, Germany) for molds and yeasts (ISO, 21527‐2, 2008), at 30°C for 120 hr in Orange Serum Agar culture medium (Merck, Darmstadt, Germany) for acid‐tolerant micro‐organisms at 30°C for 72 hr in *Lactobacillus* MRS Broth culture (Merck, Darmstadt, Germany) for heterofermentative lactic acid bacteria (ISIRI, 2017) and for *Escherichia Coli* 10 ml of diluted samples were put into double concentration of Lauryl Sulfate Broth culture (Merck, Darmstadt, Germany) and incubated at 37°C for 24–48 hr (Yörük, 2018).

### 2.4 Physicochemical properties

#### 2.4.1 pH measurement

The pH values of mayonnaise samples were measured at temperature of 20 ± 0.5°C, using a Metrohm pH meter (Model 622, Switzerland), which three replicate readings (three different samples) were taken for each pH of mayonnaises, the maximum allowed pH value for low‐fat mayonnaise during the shelf life (6 months) must be less than 4.1 (ISIRI, 2017).

#### 2.4.2 Acidity measurement

Two hundred ml of distilled water was added to 15g of the mayonnaise sample, then was mixed with 3–4 drops of phenolphthalein and titrated with NaOH 0.1 N until the first color change to violet and calculated with the corresponding formula.

Following the National Standard of Iran (ISIRI, 2017), the minimum allowed acidity values for low‐fat mayonnaise during the shelf life (6 months) must be less than 0.6.

### 2.5 Microstructure

Morphology of the mayonnaise samples microstructure was evaluated by Scanning Electron Microscopy (*SEM*, JEOL JSM‐840A, Japan) at 1, 60, 120, and 180 days of the shelf life with 250x, 500x, 1000x, and 2500x magnification level following the method described by Karami et al. (2008) and Karami et al. (2009). In this method, to prevent any damages to fat globules, mayonnaise samples were immersed in liquid nitrogen until they were freeze‐fractured. Then, the samples were coated with gold for 300s in a sputter coater (Type SCD 005, Bal Tec Inc., Balzers, Switzerland) before analysis, to avoid particle deterioration that may be caused by the electron beam. Then, immediately the samples were imaged without additional sample drying. Using an image analysis software (ImageJ, National Institutes of Health, Bethesda, Maryland, USA), the 3D structure of the samples was analyzed.

### 2.6 Statistical Analysis

Experiments were performed at four different times with three replications for each treatment. The involved factors were processing type (with preservatives, ultrasound, control) and sampling time (0, 60, 120, and 180 days). A one‐way analysis of variance (ANOVA) was performed using Minitab software (16.0) with a significance level of 0.01. Furthermore, Fisher's exact test (LSD) was used to compare the significance of differences of all analyzed data. All results were averaged and presented as means ± *SD* (standard deviation).

## 3 RESULTS AND DISCUSSION

### 3.1 Investigation of the effect of ultrasonic treatments on the micro‐organisms of mayonnaise

Survival of pathogenic bacteria in acidic conditions may pose a significant food safety challenge and should be assessed when developing new food products. No significant differences were observed between *Escherichia Coli* and heterofermentative lactic acid bacteria among all of the treatments. In all of the samples, these two bacteria were not observed.

**FIGURE 1:**
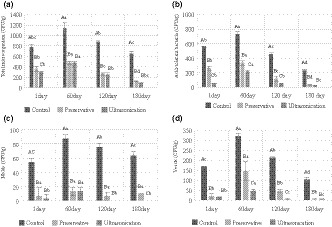
Total micro‐organisms (a), acid‐tolerant bacteria (b), molds (c), and yeasts count (d) (CFU/g) of mayonnaise samples after 1, 60, 120, and 180 days by ultrasonication and preservative agents. The results are the means, and error bars are the standard deviation of the means of three replicates, different small letters show a statistically significant difference (*p*˂ 0.01) within types of treatment durations, and capital letters show a statistically significant difference (*p*˂ 0.01) between levels with different treatments

Figure 1 shows that the US treatment reduced the population of micro‐organisms in mayonnaise during storage compared to the samples containing preservatives in their formulation. The mentioned treatment was more effective compared to the control samples, which showed a significant decrease in the population of micro‐organisms. The graph also shows the reduction of micro‐organisms’ load by adding preservatives to the samples. Also, it revealed that the highest count of micro‐organisms belongs to the control samples, which occurred on the 60th day. The lowest count of micro‐organisms was at the 180th day in ultrasonicated (US) samples. The initial populations of total micro‐organisms were 774 CFU/g in control group, 291.67 CFU/g in US treated, and 361.67 CFU/g in preservative added samples. At the 60th day, the count of micro‐organisms increased to 1,135 CFU/g in the control group, 478 CFU/g in the US samples, and 479.33 CFU/g in the preservative added samples and then gradually diminished until 180th day (654 CFU/g in control, 291.67 CFU/g in the US treated, and 361.67 CFU/g in preservative added samples). According to Figure 1, the results showed that in general, the process was ongoing for acid‐tolerant bacteria, yeasts, and molds, so that the population of micro‐organisms increased from day 1 to 60 but declined from day 60 to 180. The reason for this pattern might be the antagonistic microbial interactions during the shelf life. Cameron et al. (2010) showed that ultrasonication (20 kHz, 750 W) for 10 min decreased an initial microbial load of 1 × 10^4^ CFU/mL in milk containing 4% fat and to zero in normal saline. An acid‐tolerant micro‐organism such as lactobacilli may lead to undesirable changes in the food products and have adverse effects on sensorial properties and shelf life (Fialová et al., 2008). Predominant the *lactobacillus* species causing spoilage of mayonnaise are *Lb. brevis, Lb. casei, Lb. plantarum*, *Lb. buchneri*, and *Lb. fructivorans* (Cameron et al., 2010; Fialová et al., 2008; Manios et al., 2014). Fialová et al. (2008) reported the role of preservatives on mayonnaise‐derived lactobacilli. They studied noncontaminated and contaminated mayonnaise with 2.7 and 1.5 log CFU/g initial lactobacilli loads and showed that the lactobacilli increased to 5.4 log CFU/g in noncontaminated mayonnaise, while it reached to 7.5 log CFU/g in contaminated ones; also, the pH of the mayonnaise decreased during the shelf life. In our study, the initial population of acid‐tolerant micro‐organisms was 544.67 CFU/g in the control group, 55 CFU/g in the US treated, and 261.67 CFU/g in preservatives containing samples. These values increased until 60th day (728.33 CFU/g for control, 214.67 CFU/g for the US treated, and 337.33 CFU/g in preservative added samples) and then gradually diminished until 180th day (231.67 CFU/g in control, 20 CFU/g in the US treated, and 37.33 CFU/g in preservative added samples).

The most spoilage flora of mayonnaise and dressings are acetic acid‐resistant yeasts such as *Z. bailii* and *Pichia membranaefaciens*. These micro‐organisms can grow in the presence of a 3% acetic acid medium and weak acids medium. Some acids such as sorbic and benzoic are not effective against *Z. bailii*. The main yeast species of mayonnaise spoilage are *Z. rouxii*, *Saccharomyces cerevisiae*, and *Candida magnolia*. Other yeasts can spoil the mayonnaise including *Issatchenkia orientalis, C. inconspicua, C. parapsilosis, S. exiguous, S. dairensis, Debaryomyces hansenii, Rhodotorula mucilaginosa*, and *Torulopsis cutaneum* (Chaleshtori et al., 2018). The results (Figure 1) showed that the initial load of yeast was 168 CFU/g in control, 15.33 CFU/g in the US treated, and 21 CFU/g in preservative added samples and these values increased until 60th day (320.33 CFU/g for control, 47 CFU/g for the US treated, and 146.67 CFU/g in preservative‐containing samples) and then gradually diminished until 180th day (10.54 CFU/g in control, 5.77 CFU/g in US treated samples, and 5.77 CFU/g in preservative‐containing samples).

Molds rarely deteriorate the acetic acid containing Mayonnaise (Pitt & Hocking, 2009). The amount of acetic acid is a critical factor, somehow the acetic acid ≥ 0.5% inhibits the grow of most molds. The mold growth is limited because of limited oxygen in the packaged material. Some molds may be responsible for spoilage of the mayonnaise, such as *Geotrichum* spp., *Monascus ruber, M. acetoabutans*, and *Penicillium glaucumi*. As determined in previous studies, frequently, a selected group of micro‐organisms that were isolated from spoiled mayonnaise and similar products were lactobacilli. An essential factor which exists in mayonnaise and prevents the growth of most micro‐organisms is acidic condition. According to the above‐mentioned chart (Figure 1), the initial population of molds was 54 CFU/g in control, 3.33 CFU/g in the US treated samples, and 6.66 CFU/g in preservative‐containing samples, as these values increased until 60th day (87.33 CFU/g for control, 13.33 CFU/g for the US treated samples, and 13.33 CFU/g in preservative‐containing samples) and then gradually diminished until 180th day (63.33 CFU/g in control, 0 CFU/g in US treated samples, and 10 CFU/g in preservative‐containing samples).

Previous researchers studied the inactivation of micro‐organisms by ultrasound. They figured out the related mechanism of ultrasound on the disruption of micro‐organisms, which has been explained by acoustic cavitation and its physical, mechanical, and chemical effects that inactivate bacteria and de‐agglomerate bacterial clusters or flocks (Joyce et al., 2003). Similar results were reported in previous studies (Bi et al., 2020). They investigated the potential of using US combined with lysozyme (US + Lys) treatment as a pasteurization technology in liquid whole egg processing. Their results showed that the inactivation of *S. typhimurium* by the US and US + Lys enhanced by increasing the treatment temperatures, US power, and time. Sarkinas et al. (2018) reported that decontamination of micro‐organisms influenced by the power of ultrasonic waves, exposure time, and bacteria type. They found that gram‐positive bacteria (*B. cereus* and *L. monocytogenes*) are more susceptible to the ultrasonic treatment than the gram‐negative ones (*E. coli* and *S. typhimurium*). Gao et al. (2014) discovered that the D_values_ of *Enterobacter aerogenes* to the US were 45, 14, and 11 min when cells were sonicated at 20 kHz and 0°C at 4, 8, and 12 W, respectively. Furthermore, Ngnitcho et al. (2018) discovered that *Listeria monocytogenes* on sprouts was inactivated by 3.93 and 4.47 log_10_ cycles when treated with the US at 40 kHz (400 W) and 30°C for 15 min and 40°C for 15 min, respectively.

### 3.2 Evaluation of ultrasonic treatments on the physicochemical properties of mayonnaise

Statistical analysis showed that there was a significant difference between the pH and acidity of US treated and preservative‐containing low‐fat mayonnaise samples during storage from day 1 to 180 at 4°C (Tables 2 and 3).

**TABLE 2:** Comparison of treatments averages in terms of pH

Treatment	Day 1	Day 60	Day 120	Day 180
Control	3.75 ± 0.01^Bbc^	3.86 ± 0.00^Ba^	3.76 ± 0.00^Bb^	3.74 ± 0.005^Bc^
With preservative	3.86 ± 0.01^Ab^	3.96 ± 0.01^Aa^	3.87 ± 0.01^Ab^	3.81 ± 0.01^Ac^
US treated	3.72 ± 0.02^Ca^	3.74 ± 0.01^Ca^	3.62 ± 0.02^Cb^	3.56 ± 0.04^Cc^

Note

Numbers in each *column* with different *capital letters* based on Fisher's exact test (LSD) showed a statistically significant difference (*p* ˂ 0.01).

Numbers in each *row* with different *small letters* based on Fisher's exact test (LSD) showed a statistically significant difference (*p* ˂ 0.01).

**TABLE 3:** Comparison of treatments averages in terms of Acidity

Treatment	Day 1	Day 60	Day 120	Day 180
Control	0.64 ± 0.01^Cb^	0.56 ± 0.04^Bc^	0.70 ± 0.01^Ba^	0.73 ± 0.01^Ba^
With preservative	0.67 ± 0.02^Ba^	0.58 ± 0.017^Bab^	0.67 ± 0.02^Bab^	0.64 ± 0.01^Cb^
US treated	0.80 ± 0.005^Ac^	0.66 ± 0.20^Ad^	0.90 ± 0.01^Ab^	0.95 ± 0.01^Aa^

Note

Numbers in each *column* with different *capital letters* based on Fisher's exact test (LSD) showed a statistically significant difference (*p* ˂ 0.01).

Numbers in each *row* with different *small letters* based on Fisher's exact test (LSD) showed a statistically significant difference (*p* ˂ 0.01).

Acidity is the most important intrinsic characteristic of mayonnaise, dressings, and sauces in determining the growth and the survival of pathogenic bacteria, followed by the salt and sugar content which play minor roles. Still, they have an interactive effect with acetic acid in vinegar on inhibiting the growth of foodborne pathogens. The more significant antimicrobial effect of mayonnaise relates partially to its lower pH and the lysozyme in the egg white used in the formulation (Aganovic et al., 2018; Chaleshtori et al., 2018). In Table 2, the Fisher test showed a statistically significant difference between on pH of the control samples compared to the treated ones. Accordingly, the mean of pH in mayonnaise samples containing preservative was highest during storage. Also, the control samples had a higher pH compared to the US samples. As a consequence, pH in all samples increased from the first day until the 60th day and decreased from 60th to the 180th day. The highest pH was 3.96, which was observed in the samples containing preservatives that occurred on the 60th day, and the lowest pH was 3.56 for the US treated samples, which occurred on 180th day.

The results represented in Table 3 showed that the US treated samples had the highest acidity compared to the control and preservative‐containing samples; also, the highest acidity value was 0.95 that related to the US treated on 180th day. Overall, the acidity of all samples reduced from the first day to the 60th day and increased from 60th to 180th day. Acetic acid is the predominant acid in mayonnaise, which presents as various kinds of vinegar. The effects of acidity on micro‐organisms include the following: (i) the effect of pH alone, (ii) the impact of undissociated forms of a particular acid, and (iii) the specific effects of organic acids (Jalilzadeh et al., 2018). They reported that the pH values of all sonicated samples of Iranian ultrafiltered feta‐type cheese were lower than that of the control at the end of the storage period. Also, they found that the acidity of all sonicated cheese samples was higher than control group samples on 60 days of ripening, and there was no linear relationship between sonication frequency and acidity, increasing the acidity due to ultrasound can be attributed to the hydrolysis of triglycerides and the consequent increase of free fatty acids (FFA) (Uluko et al., 2015). Bermúdez‐Aguirre et al. (2009) reported that ultrasound pretreatment with different amplitudes and times significantly (*p* <.05) decreased the pH of milk, which is similar to the findings of the present study.

### 3.3 Scanning electron microscopy for microstructure of mayonnaise

**FIGURE 2:**
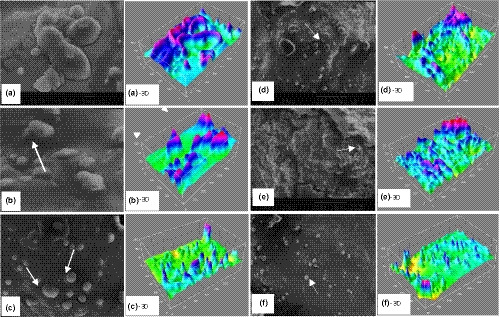
*SEM* images of flocculation and aggregation of oil droplets in the microstructure of mayonnaise samples 1 (a: Control, b: with preservative, and c: treated by sonication), and 60 days (d: Control, e: with preservative, and f: treated by sonication) after production. Tri‐dimensional (3D) images of *SEM* micrographs are shown behind the original images

**FIGURE 3:**
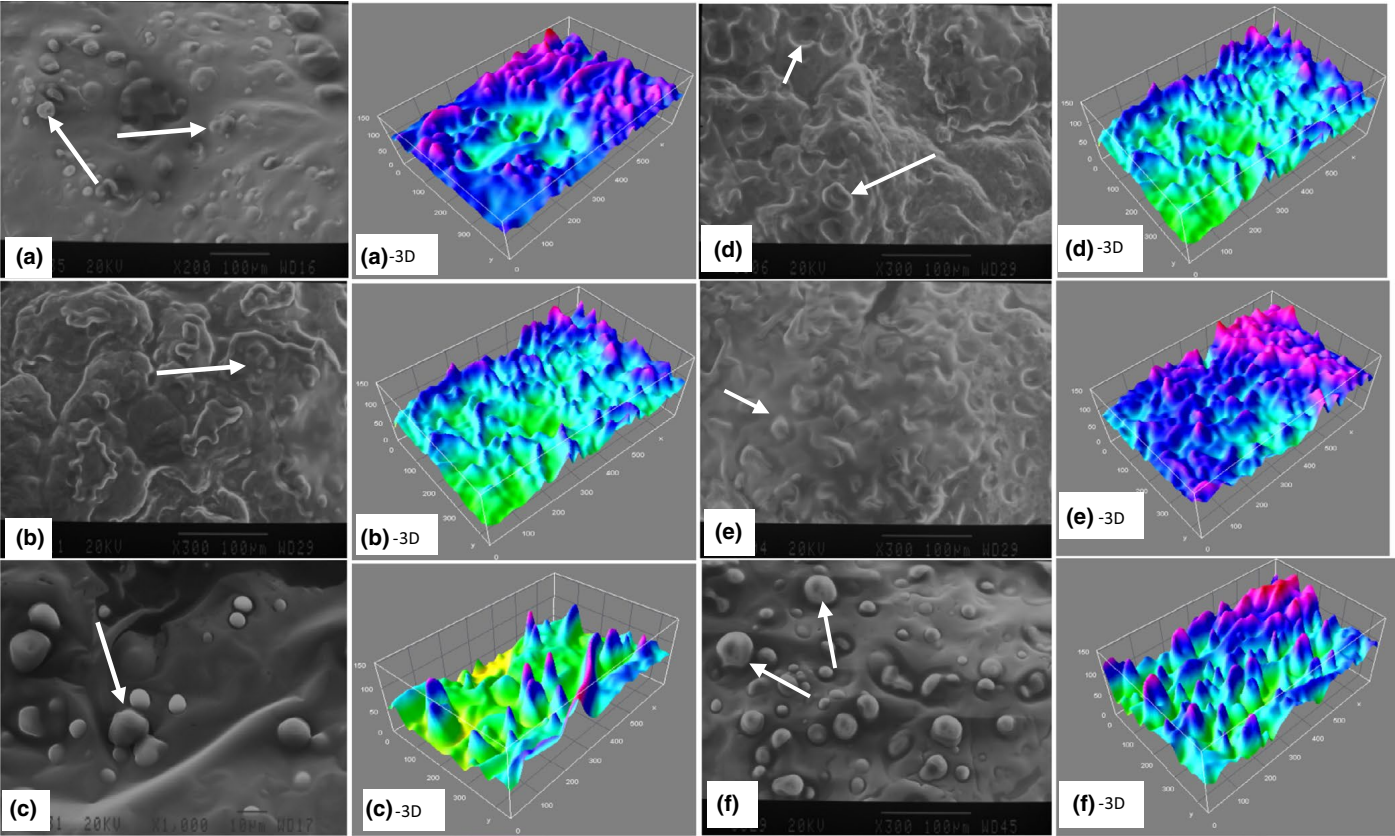
*SEM* images of flocculation and aggregation of oil droplets in the microstructure of mayonnaise samples after 120 (a: Control, b: with preservative, and c: treated by sonication) and 180 days (d: Control, e: with preservative, and f: treated by sonication) of production. Tri‐dimensional (3D) images of *SEM* micrographs are shown behind the original images

The microstructure of mayonnaise samples was observed by *SEM* micrographs, to identify the dispersion characteristics of oil droplets and protein aggregates. The images obtained by *SEM* of the mayonnaise samples during storage time (1, 60, 120, and 180 days) are shown in Figures 2 and 3 (a–f), and Tri‐dimensional (3D) images of the *SEM* micrographs are presented behind the original (two‐dimensional, 2D) images. Three micrographs were taken from each mayonnaise sample, and conclusions were similar among all replicates (only representative micrographs were selected for presentation). These microscopical images indicated that fat is present in the mayonnaise in different forms and sizes.

Fat plays an essential role in the characteristic flavor, texture, and acceptability of mayonnaise. The results of the current study showed that (Figure 2) in the control sample (a and d) and with preservative samples (b and e), the oil droplet size was more extensive, and the shape of the particles was rough after 1–60 days. Figures (c and f) represents a set of microstructures of the mayonnaise exposed to sonication treatments, the obtained *SEM* images, in comparison with control samples, exhibited more homogenous structures and fragments. These might be generated because of the cavitational forces exerted by the probe during ultrasonication and resultant microstreaming and destructive forces. Aganovic et al. (2018) reported the oil droplet size distribution and microscopic imaging of the Ultra‐high‐pressure homogenization (UHPH) emulsion using confocal laser scanning microscopy (CLSM). They revealed more even distribution of components after UHPH process, compared to its commercial full‐fat and low‐fat counterparts. Ultrasonication is an excellent way to produce physically stable emulsions and avoiding sedimentation (Campoli et al., 2018).

As shown in Figure 2 (d–f), the microstructure of mayonnaise samples showed significant differences 60 days after production. Increasing of the shelf life led to the progressive accumulation and aggregation of fat globules (impart smooth mouth‐feel just like spherical rollers) (D and E). The internal phase obviously aggregated, and some oil droplets were connected to each other. Further aggregation of protein particles leads to formation of larger sized aggregates. As showed in Figure 2 (f), related to ultrasound, all samples behaved smooth surface appearance, indicating the regular internal network with no unacceptable excessive aggregation and flocculation. Also, besides the identifiable spherical structure of egg white microparticle became less and smaller in the US samples, indicating the fragile property of the protein particles, which can be disrupted easily after US and may not produce firm sensible powder in the mouth. The formation of connections between droplets can have an impact on the textural behavior of the product (Langton et al., 1999). Tri‐dimensional images can reflect the appearance of the prepared mayonnaise samples more intuitively. Since oil‐in‐water emulsion is a thermodynamically unstable system, proteins with higher surface hydrophobicity and smaller size, preferred to adsorb on the interface, which reduce the tension between the two immiscible liquid. Ma et al. (2019) studied the subsequent ultrasonic effects on the stability of oil‐in‐water emulsions. They found that it can be a potential tool to modify emulsifying characteristics of the Cod protein.

The results represented in Figure 3 (a–c) indicated that the fat globules of mayonnaise samples after 120 days of production surrounded by the porous structure of protein network. At this point, the oil droplets got closer and closer together, and the oil droplets fluctuated (a and b). The oil droplets in Figure 3 (a and b) were nonuniform, and they were less uniformly dispersed in this system. The internal phase of fat molecules was aggregated, and some oil droplets were attached. It was reported that generally, excellent product stability was not only associated with small oil droplet, but also with uniform oil droplet diameter (Ma et al., 2019). The sample prepared under sonication had similar number and sized white spherical particles (Figure 3c). The pictures also verified that treating by sonication led to soft matrix phase with visible spherical‐like appearance that is a critical contributing factor to cavitation damage of the physical properties. Further, food‐based emulsions made with flaxseed oil and skim milk using ultrasound have shown stability for up to 7 days without requiring the addition of an external emulsifier [48].

In the original *SEM* images of Figure 3 with showed magnifications (d–f), the black arrows indicate that interspace voids represent the oil droplets (d and e), while the network structures correspond to the continuous aqueous phase in the emulsion. The oil droplets move toward the mayonnaise surface according to the Stokes' law. The interspace voids were more abundant, in the D and E compared with the F sample, so exhibited larger oil droplets. This phenomenon provided evidence for the previous speculation on the changes in viscosity and creaming specifications. However, the samples (F) behaved a more compact structure with indistinguishable oil droplets. Zhou et al. (2018) also found that ultrasound treatment led to an increase in emulsion activity index and emulsifying stability of walnut protein. They explained that smaller droplets were formed in the sonicated samples, the reason for that was a higher surface hydrophobicity of treated protein particles interfaces between water and oil phase.

In Summary, the obtained images suggest that treatment by sonication may induce stronger interaction between oil droplets and granular protein. That means during shelf life of product, oil droplets accumulate much less, even on day 180, which expires date of this kind of low‐fat mayonnaise according to Iranian standards. Therefore, microbial and chemical spoilage occurs much later. According to the images of preservative‐containing samples during the shelf life, rapid accumulation of oil droplets occurred, and there was no significant difference compared to the control samples. So, preservatives may prevent microbial spoilage until day 180, but they are not effective against chemical decomposition of light mayonnaise samples.

## 4 CONCLUSION

This study demonstrated that pretreatment with sonication at 20 kHz and 750W significantly reduced the growth of micro‐organisms in low‐fat mayonnaise. Also, the pH values of all treated samples at the end of storage were lower than that of control samples and the preservative‐containing samples, so the highest acidity was found in the sonicated samples. The obtained *SEM* images showed that sonication had a significant effect on the microstructure and texture of low‐fat mayonnaise and induced conformational enhancement of the stability of subsequent oil–water emulsions. As well in sonicated samples during shelf life (6 months), aggregation of oil droplets occurs very slowly and maintains its stability within the texture compared with the preservative‐containing and control samples that during their shelf life, oil droplets quickly move toward the mayonnaise surface and leads to chemical decomposition. According to the findings of this research, on the harm of preservatives, ultrasound can be an excellent technique to replace these carcinogens without any adverse effect on texture and bacteria. It was concluded that ultrasonication is a safe method to produce a preservative‐free mayonnaise.
